# Collective properties of *Petitella georgiae* in tube environments

**DOI:** 10.1038/s41598-024-78614-w

**Published:** 2024-12-02

**Authors:** Shuang Gu, Quan Quan

**Affiliations:** The School of Automation Science and Electrical Engineering, Beihang University, Beijing, 100191 China

**Keywords:** Fish swarm, Tube environments, Collective properties, Mathematics and computing, Behavioural ecology

## Abstract

The movement of biological swarms is widespread in nature, and collective behavior enhances a swarm’s adaptability to its environment. However, most research focuses on free swarm movement, overlooking the impact of environmental constraints such as tubes. This study examines the swimming behavior of *Petitella georgiae* through a tube. Observations of position, speed, and direction reveal that each fish is influenced by the swarm’s distribution in its field of view. The speed ratio between the *middle region* and *edge region* positively correlates with tube angles, and higher speeds are associated with higher densities within specific angle ranges.

## Introduction

Biological swarm behavior exists widely in nature, such as food transport of ant colonies^1^, forest hunting of wolves^2^, and collective motion of fish swarms^3^. The apparently regular swarm behaviors in these animals make it feasible to extract relevant underlying mechanisms. This visibility presents opportunities for deeper exploration of the properties driving such collective phenomena.

In terms of criteria for the operation of a biological swarm, the movement of individuals in the Vicsek model was updated according to all neighbors’ movement, and many studies were carried out according to this classical model^4–7^. The main conclusion of the Vicsek model was that swarm behavior exhibited self-organization and coordination, and interaction among individuals could lead to orderly movement of the swarm. This model revealed that in the absence of a centralized leader, simple interaction among individuals could produce collective properties, such as mutual alignment and collective movement. For instance, birds fly together and constantly change relative positions, but there is no leader to make overall planning. Reaction among individuals, which helps spread information in the swarm, plays a vital role in achieving unified swarm movement.

Since the 1970s, biological swarm has become an essential part of experimental research, and researchers have applied numerous methods when studying biological swarms. Herbert-Read^8^ introduced the utilization of computer vision and automated tracking technologies to collect extensive trajectory data on animal swarms. It was detailed how the data can be analyzed to infer the underlying rules of individual movements within these swarms. As to specific studies, the team collected female mosquitofish (*Gambusia holbrooki*) and maintained them in 50 L aquaria on a 12:12 hour, dark:light photo period for observation^9^. Couzin et al.^10^ conducted experiments on swarms of *golden shiners*. Their tracking was achieved through a two-step process^11^: raw images were converted to grayscale and inverted to identify fish through thresholding and edge detection, allowing for the calculation of their geometric centers and areas. In the second step, fish were tracked across frames by matching objects using a MATLAB implementation of an algorithm by Sbalzarini and Koumoutsakos, with custom software compiling these matches into complete trajectories. Jiang et al.^12^ used a data-driven analysis method with short-term directional correlations to investigate how *Hemigrammus rhodostomus* respond to their neighbors during collective movement, particularly in U-turn events. Lopez et al.^13^ used tubes to track fish movement, and three algorithms (MOSSE, Seq-NMS and SiamMask) were used to analyze the fish. Kane et al.^14^ placed fish in a tank and performed analysis on collected data, including velocity, total line distance, angular change, space utilization, and fractal dimension.

On the analysis of the biological swarm coordination mechanism, Georgopoulou et al.^15^ studied the emergence and repeatability of coordinated motion in stickleback fish swarms. They found that initially uncoordinated swarms quickly developed leader-follower roles, which were consistent across trials. This rapid coordination and role stability may help reduce uncertainty in the fish’s social interactions, particularly in dynamic environments with frequent group changes. Chen et al.^16^ analyzed high-resolution GPS data from homing pigeon flocks to investigate their movement patterns. The results revealed that while a pigeon flock maintains a long-term positional leader during smooth, continuous trajectories, leadership temporarily shifts to a different pigeon during sudden turns or zigzag movements. Ashraf et al.^17^ used *Petitella georgiae* with high cohesion to analyze the swarm movement of fish. It was observed that when fish were compelled to swim at high speeds, which significantly exceeded usual free-swimming velocity, swarm formation would take the form of a “phalanx” or “orsoldier”. Zhou et al.^18^ proposed a novel order parameter and a measurement framework for group structures. In this fellow-following principle, the study object would adjust its movement to maintain a proper distance from the other objects. Gomez et al.^19^ studied the spontaneous behavior of small sheep flocks and summarized the leader-follower coordination mechanism.

**Fig. 1:**
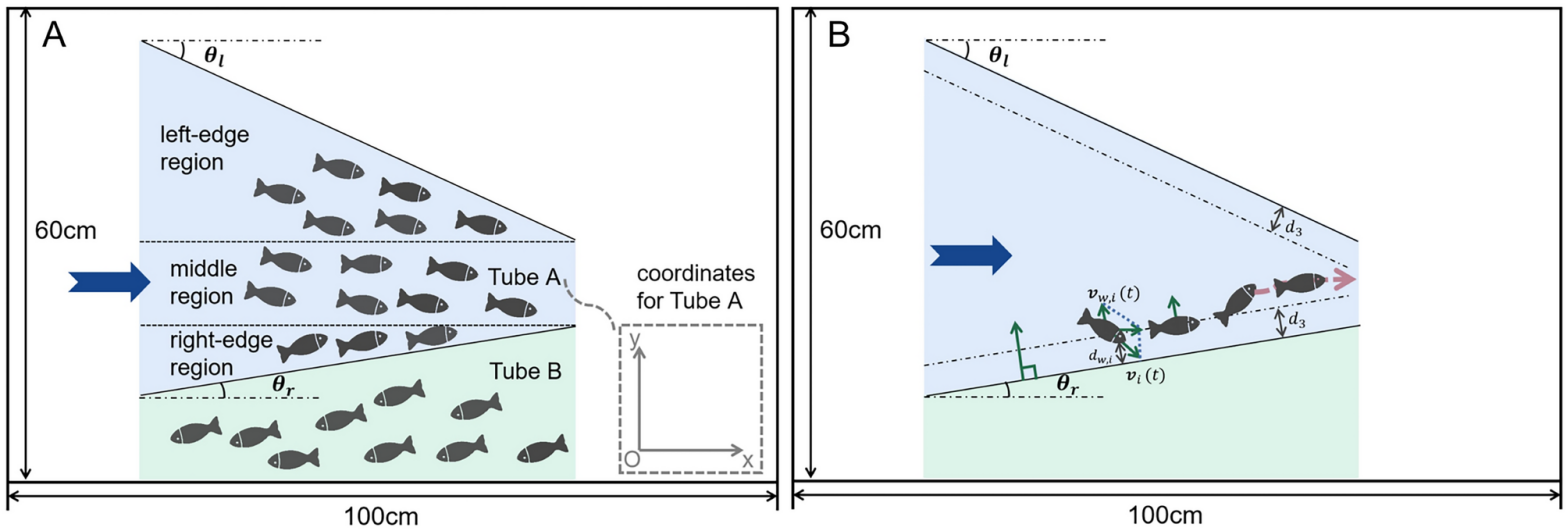
This is a top view of the rectangular fish tank with corresponding dimensions. Two acrylic panels form three tubes marked by arrows to allow the fish swarm to pass through. (**A**) The division method of different regions. (**B**) The collision avoidance process.

The work done to produce the results in this paper includes:

1. An experimental system was established for the fish swarm, incorporating eight tubes with varying angles for the fish to navigate. The species used in this study was *Petitella georgiae*, known for its ability to swim spontaneously in swarms under natural light conditions. In this paper, the interior of the tube was divided into *middle region* and *edge region*, as illustrated in Fig. 1A. The trapezoidal tube’s upper base and height define *middle region*, and the two triangular sections constitute the *edge region*. Specifically, for Tube A in Fig. 1A, two edge regions are identified: the *left-edge region* and the *right-edge region*.
2. The software processing module utilized Zootracer (Microsoft Corporation, 2014) and MATLAB for analysis. Initially, the video depicting the swarm behavior was processed in Zootracer, breaking it down into individual frames. Subsequently, Zootracer identified the trajectories and generated coordinate data in two-dimensional .csv format. Movement parameters, including speed, direction, and angles, were then calculated using MATLAB.
3. In the property analysis module, we analyzed the influence of tubes with varying inclination angles on the movement of the fish swarm using data obtained from the software calculations. Three properties were developed to represent swarm traffic, and dynamic simulations were conducted based on these properties.

## Material and methods

The objective of the experiment was to record the swarm behavior of fish within tubes, collect movement data, and analyze the properties of biological cooperation using specialized software, so series of experiments were conducted. This section outlines the design and construction of the experimental platform, the selection of fish species, and the methodologies employed for data acquisition and analysis.

### General design

In this experiment, a constrained environment was constructed from scratch. Rather than allowing fish to swim freely in a rectangular tank, we designed a trapezoidal tube to focus on observing swarm behavior at the tube’s entrance. The spontaneous swarm phenomenon serves as the foundation for our experimental verification. Additionally, quantitative analysis was conducted using Zootracer and Matlab software, so as to reach the identification of three properties. These properties can be incorporated into modular codes to generate dynamic simulation.

### Fish and environment

*Petitella georgiae* has a body length of 3.5–4 cm and a height of 0.5–0.6 cm. They were procured from a local aquarium supplier and reared in a 360-liter aquarium tank, measuring 100 cm in length, 60 cm in width, and 60 cm in height. The water temperature was maintained at approximately 25 degrees Celsius, utilizing a heating rod when necessary to create optimal conditions that foster their comfort and active schooling behavior. Such an environment is crucial for encouraging their natural tendency to swim in swarm. Additionally, the fish do not require frequent feeding. Providing commercial flake food every few days suffices to meet their nutritional needs.

### Tube configuration

**Fig. 2:**
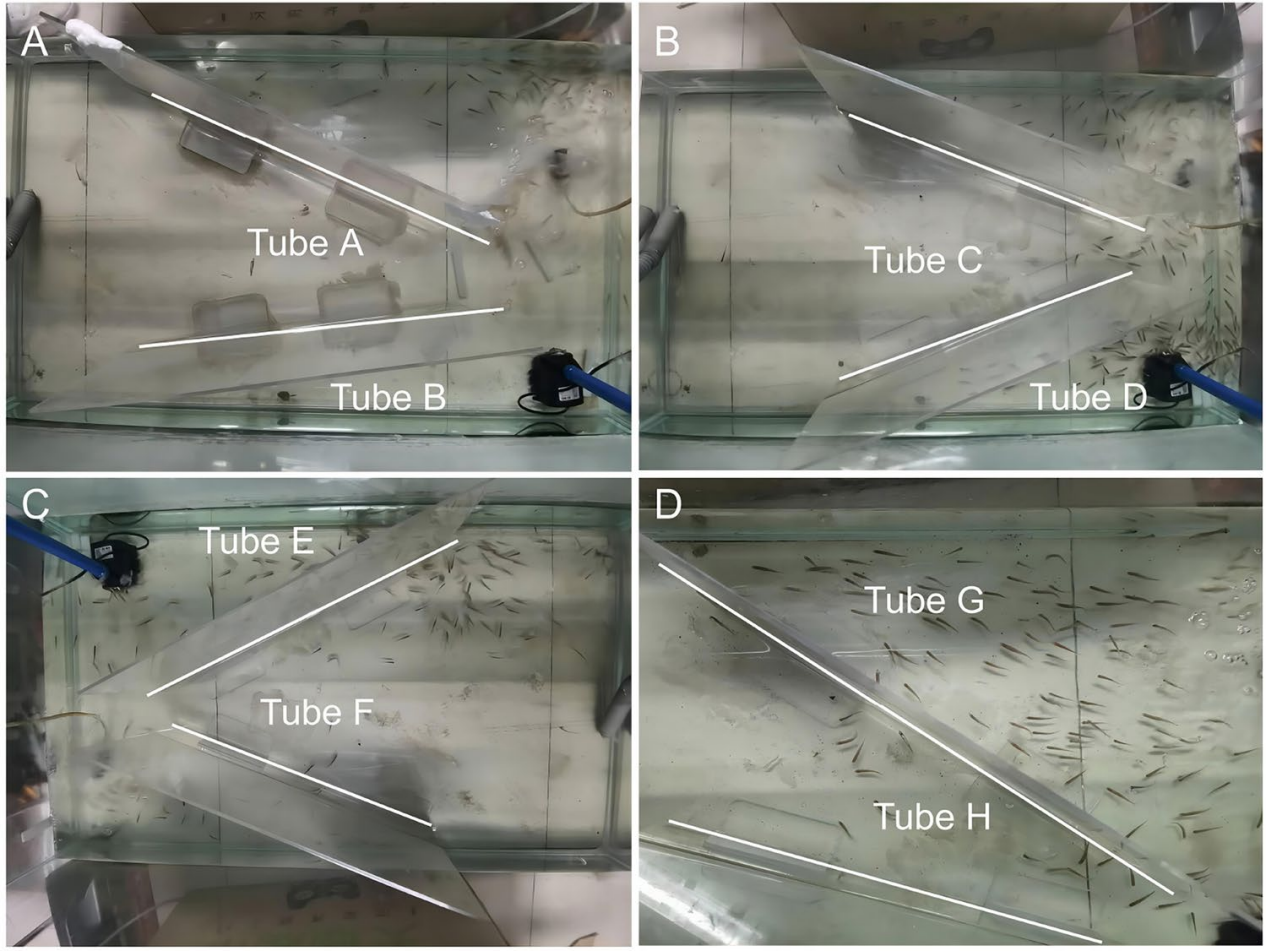
Construction of eight tubes in this experiment, from Tube A to Tube H.

In the fish tank, two vertically positioned acrylic plates were arranged to create three tubes. By adjusting the angles of these plates, tubes with varying inclinations were formed, allowing fish to swim through in crowded formations while enabling the observation of their swarm behavior. The actual appearance of the eight tubes is depicted in Fig. 2.

**Table 1:** Angles of the eight tubes.

Tube	Angle/degree	Tube	Angle/degree
Tube A	29.05	Tube B	5.52
Tube C	34.12	Tube D	22.03
Tube E	23.84	Tube F	51.00
Tube G	36.32	Tube H	19.48

In this study, the eight tubes are sequentially designated as Tube A through Tube H. Tube A is illustrated in Fig.1A for detailed examination. In this setup, the direction in which fish exit the tube corresponds to the x-axis, while the y-axis follows the right-hand rule. For instance, the x-axis of Tube A is indicated in Fig.1A. Notably, since the fish in Tube A and the other two tubes swim in opposite directions, their coordinates on the x-axis are oriented accordingly. The angles of the eight tubes were calculated and are presented in Table 1.

### Visualization

Spontaneous swarm behavior was continuously recorded for 24 hours a day over approximately one month, using a TP-LINK camera positioned perpendicular to the water surface of the tank. The camera operated at a resolution of 2592x1944 pixels with a focal length of 4 mm, effectively capturing the swarm behavior for subsequent analysis. The obtained images were downloaded and stored in the same root directory.

We subsequently reviewed the recorded footage and edited segments from the lengthy original videos for further analysis (Movie S1–S8). In these videos, *Petitella georgiae* exhibited distinct spontaneous schooling behavior while navigating through narrow tubes, with each useful segment lasting approximately tens of seconds.

### Experimental procedure

The total number of fish utilized in the experiment was approximately 60. Fish exhibiting erratic swimming behavior, as opposed to those demonstrating pronounced schooling behavior, were excluded from the study. This resulted in a focus on 16 fish that exhibited typical schooling behaviors, which were used for data analysis. The same group of fish was employed throughout all trials. Upon acquisition, the fish were immediately subjected to experimentation and subsequently released alive, with the entire process receiving the necessary ethical approval.

When no experimental recording was taking place, the water depth was maintained at 15–20 cm to allow for free-swimming conditions. During recording, however, some water was drained to ensure that individual fish would not overlap in the overhead camera’s field of view, resulting in only a single layer of fish being captured in the video. This setup enabled the fish swarm to maintain orderly and efficient coordination, without congestion throughout the process.

During the schooling behavior, no external stimuli, such as light or food, were used to induce the phenomenon. This species of fish is not particularly sensitive to food, and the schooling behavior could occur naturally under natural light conditions, without the need for artificial lighting. We actually made several attempts with other species before this experiment, including *Goldfish* and *Danio rerio*, as well as attempts to guide their behavior with light stimuli, were not particularly successful. The experiment demonstrated that the fish swarm could spontaneously exhibit the expected schooling behavior.

Videos from the experiments presented in this paper (Movies S1–S8) were analyzed using Zootracer software, and statistical data of the fish swarm behavior were collected. The original .csv files, along with calculated statistics on speed, position, and direction (Datasets S1–S9), are provided. Additionally, MATLAB codes for data analysis and figure generation are included.

## Data analysis

In this paper, the area within a tube was divided into *middle region* and *edge region*. The upper base and the height of the trapezoid tube formed *middle region*, and the remaining two triangles formed *edge region* (Fig. 1). For tubes in different shapes, the calculation process was also different. For a tube composed of two acrylic panels, there existed two *edge regions*, and they were named *left-edge region* and *right-edge region*.

**Table 2:** Parameters of the 16 fish at frame 37 in Tube A.

Number	X-axis	Y-axis	Speed-x	Speed-y	Speed
1	1070.00	618.00	7.00	-8.00	10.63
2	1905.00	1078.00	0.00	0.00	0.00
3	1132.50	477.00	6.50	-1.00	6.58
4	1073.00	612.00	3.00	-5.50	6.26
5	1247.00	517.00	11.50	2.50	11.77
6	1242.00	502.00	9.50	0.50	9.51
7	1905.00	1078.00	0.00	0.00	0.00
8	905.40	553.20	8.40	-0.80	8.44
9	1264.00	553.00	641.00	525.00	0.00
10	985.75	539.50	5.25	-2.50	5.81
11	1144.00	569.00	6.50	0.00	6.50
12	1263.00	469.00	5.00	1.50	5.22
13	1223.50	579.50	11.50	3.50	12.02
14	869.80	688.40	6.60	-2.20	6.96
15	989.00	540.00	5.00	0.00	5.00
16	1139.00	605.00	29.50	-2.00	29.57

The data obtained after fitting was loaded into Matlab for extraction (Movie S9). For the movie of each tube, this paper extracted the speed and position of 16 fish at frames with the same intervals. For instance, for the moment of Frame 37 in the movie of Tube A (Movie S1), the extracted data is present in Table 2. As shown in Dataset S4, there were 18 frames chosen in Tube A, 10 in each of Tube B, C, E and G and 7 in each of Tube D, F and H. That is to say, there were 79 tables in the form of Table 2 for analysis in all.

In Table 2, the column “Number” records the number of each of the 16 fish for research. Columns “X-axis” and “Y-axis” record their position in the coordinate system for Tube A at Frame 37 of the movie (Movie S1). The column “Speed-x” records the absolute value of velocity in the x-axis at this moment, and “Speed-y”is in the y-axis. The column “Speed” records the magnitude of the combined value of the velocities in both directions.

**Table 3:** Speed and location in middle region and edge region in Tube A.

frame	${V_{middle}}$	${V_{edge}}$	Ratio-s	${N_{middle}}$	${N_{edge}}$	Ratio-n
1	22.70	22.07	1.03	10.00	6.00	1.67
10	15.89	12.51	1.27	11.00	5.00	2.20
19	12.61	11.59	1.09	11.00	5.00	2.20
28	13.02	10.47	1.24	11.00	5.00	2.20
37	8.28	7.42	1.12	12.00	4.00	3.00
46	9.13	4.77	1.91	11.00	5.00	2.20
55	6.43	0.39	16.63	8.00	8.00	1.00
64	8.65	0.39	22.27	6.00	10.00	0.60
73	10.16	0.16	63.93	3.00	13.00	0.23
82	3.86	0.16	24.30	3.00	13.00	0.23
91	2.86	0.08	35.47	3.00	13.00	0.23
100	1.88	0.07	25.12	2.00	14.00	0.14
109	1.88	0.12	15.60	2.00	14.00	0.14
118	5.10	0.12	42.34	2.00	14.00	0.14
127	1.93	0.47	4.14	1.00	15.00	0.07
136	7.00	0.00	0.00	1.00	15.00	0.07
145	1.14	0.00	0.00	1.00	15.00	0.07
154	0.00	0.07	0.00	0.00	16.00	0.00

Then for every of the 79 tables mentioned above, the average speed and number of the 16 fish in the two regions at the corresponding moment was calculated. Ultimately, each tube got a table through calculation, which was presented in Table 3.

In Table 3, the column “Frame” records the moment in this tube for analysis. Average speed in *middle region* is ${V_{middle}}$, and average speed in *edge region* is ${V_{edge}}$. The column “Ratio-s” records the ratio of ${V_{middle}}$ and ${V_{edge}}$. Number of fish in *middle region* is ${N_{middle}}$, and the number of fish in *edge region* is ${N_{edge}}$. The column “Ratio-n” records the ratio of ${N_{middle}}$ and ${N_{edge}}$.

After eight tables were obtained, corresponding movies were viewed to find out the segment of time during which the 16 fish met requirements. For instance, in Table 3, the period of Frame 28-46 in the movie met requirements, so the rows of statistics corresponding to Frames 28, 37 and 46 were selected. Statistics in these lines were averaged to reveal the average speed and average number in this tube, so the average speed and number of fish in *middle region* and *edge region* of Tube A were obtained. Such analysis was carried out for all eight tubes, and Table 4 was obtained.

**Table 4:** Speed analysis in middle region and edge region.

Tube	Ratio-s	Ratio-n	Angle/degree	Cosine
A	1.42	2.47	29.05	0.87
B	1.71	4.33	5.52	1.00
C	1.40	2.48	34.12	0.83
D	1.52	3.00	22.03	0.93
E	1.51	4.76	23.84	0.91
F	1.14	1.67	51.00	0.63

In Table 4, the column “Tube” records the index of each tube, the columns “Ratio-s” and “Ratio-n” are calculated in the previous step, including Table 3. The column “Angle” records the angle of each tube, and the column “Cosine” records the cosine calculated from the column of “Angle”.

**Fig. 3:**
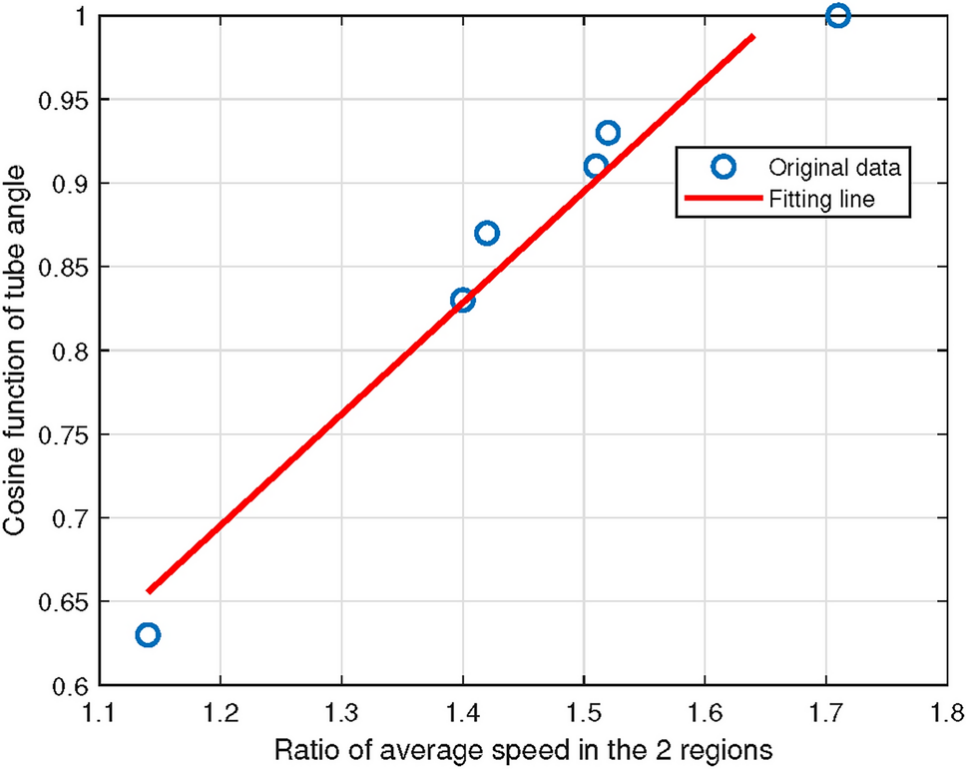
The distribution of speed ratio and cosine of tube angle, which basically fits a linear relation.

This paper applied the cosine of the inclination angle of each tube to carry out fitting analysis and found that the ratio of average speed in the two regions and the cosine of tube angle met a good linear relationship (Fig. 3), and the correlation coefficient was given in Eq. (1), so the fitting relation was gained as Eq. (1) in the following

1 $$\begin{aligned} \begin{aligned}&Corr\left( \frac{{V_{middle}}}{{V_{edge}}},{\cos \theta }\right) = 0.98,\\&\frac{{V_{middle}}}{{V_{edge}}} = 1.5033 \cdot {\cos |\theta _{r}-\theta _{l} |} + 0.1545. \end{aligned} \end{aligned}$$

**Fig. 4:**
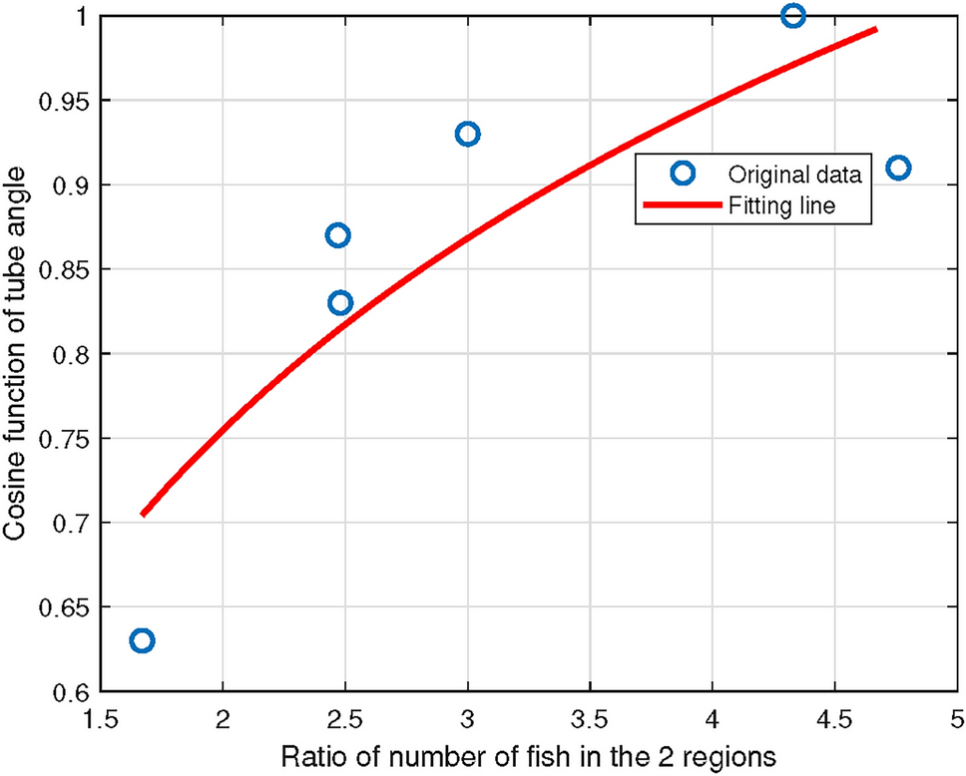
The distribution of number ratio and cosine of tube angle, which basically fits a logarithmic relation.

In this paper, *Corr* represents the correlation coefficient, which measures the strength and direction of the linear relationship between two variables. It quantifies how changes in one variable are associated with changes in another, with values ranging from $-1$ to 1. A value of 1 indicates a perfect positive correlation, $-1$ indicates a perfect negative correlation, and 0 indicates no linear relationship.

Based on the eight movie segments, this paper calculated the probability density distribution of *middle region* and *edge region*, and found that fish swarm tended to concentrate in the *middle region*. Therefore, we wanted to study the relationship between the ratio and tube angle from calculation (Table 4). It was found that they satisfied the exponential relationship (Fig. 4), and the MSE parameter was given in Eq. (2), so the fitting relation was gained as Eq. (2) in the following

2 $$\begin{aligned} \begin{aligned}&MSE\left( \frac{N_{middle}}{N_{edge}},{\cos \theta }\right) =0.0035\quad . \\&\frac{N_{middle}}{N_{edge}} = e^{{3.5971\cos |\theta _{r}-\theta _{l} |- 2.0173}}\quad .\\ \end{aligned} \end{aligned}$$

In this paper, MSE stands for Mean Squared Error, a common metric used to evaluate the difference between predicted and actual values in a model. It is calculated as the average of the squared differences between the predicted values ${\hat{y}_i}$ and the actual values ${y_i}$, as in the following

3 $$\begin{aligned} \begin{aligned} MSE = \frac{1}{n}\sum \limits _{i = 1}^n {{{({y_i} - {{\hat{y}}_i})}^2}} \end{aligned} \end{aligned}$$

Here, *n* represents the number of data points. A lower MSE indicates better predictive accuracy, as it reflects a smaller gap between the predicted and actual values.

## Collective properties

Three fish swarm properties were established based on data obtained from the experiment. Among three properties, *fish movement property in tubes* was the core property which specified the movement of every single fish in the swarm, *velocity distribution of fish swarm in tubes* and *density distribution of fish swarm in tubes* were obtained from experimental data observation, and were also set as the initial condition of the simulation. The distribution of fish under the operation of the first property also supported the verification of the other two properties.

### Property 1: fish movement property in tubes

**Fig. 5:**
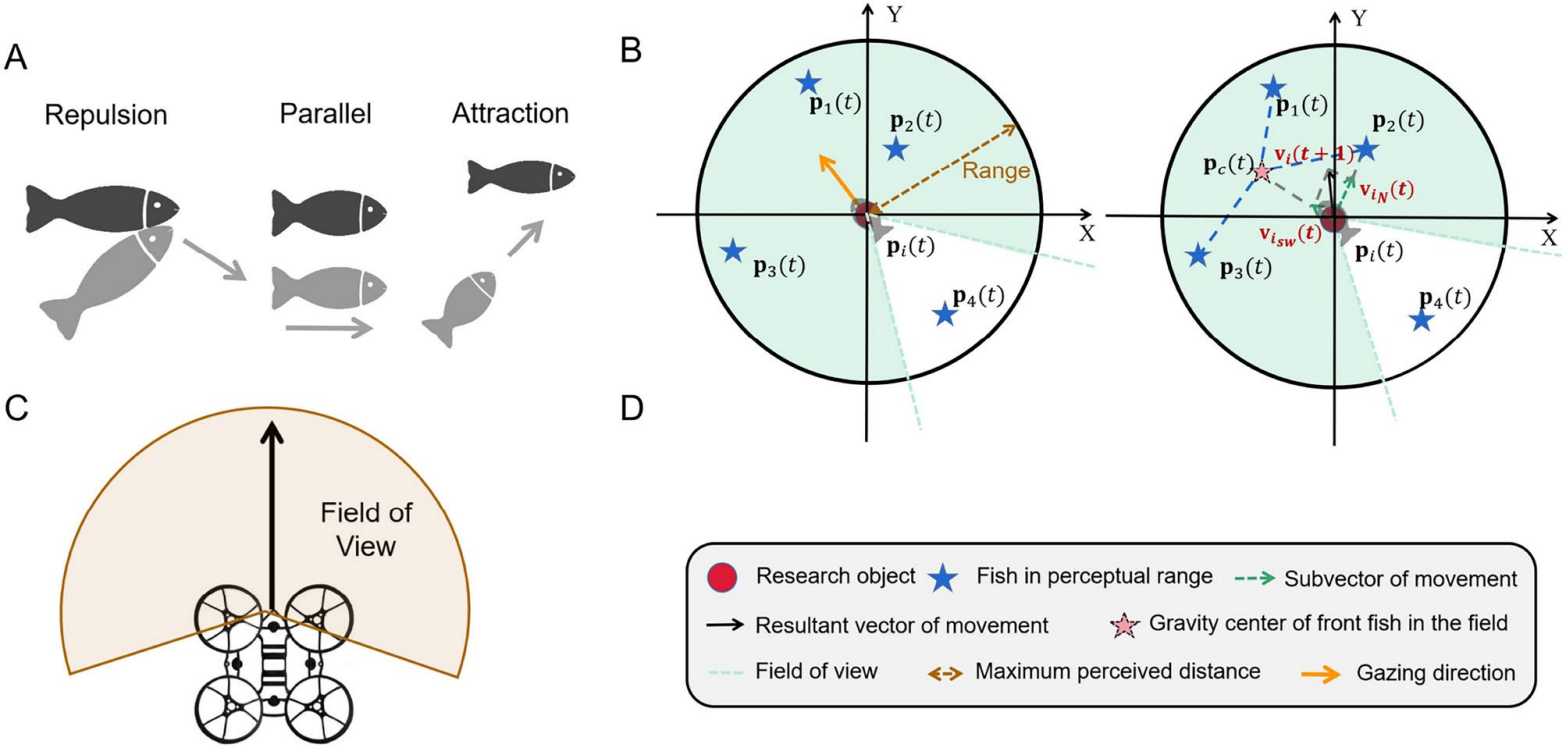
This figure corresponds to *fish movement property in tubes*. (**A**)Three different cases for $\textbf{v}_{s,i}(t)$. (**B**)*Range* represents the maximum perceived distance of the fish eye, and the green area is its field of view. Locations of $\textbf{p}_{1}(t)$ and $\textbf{p}_{2}(t)$ are within the perceptual range. Among the points, $\textbf{p}_{2}(t)$ is the the nearest fish, and $\textbf{p}_{4}(t)$ is regarded invalid. Location of $\textbf{p}_{c}(t)$ is the center of gravity of $\textbf{p}_{1}(t)$, $\textbf{p}_{2}(t)$ and $\textbf{p}_{3}(t)$. $\textbf{v}_{s,i}(t)$ is the vector pointing to $\textbf{p}_{c}(t)$, and $\textbf{v}_{n,i}(t)$ is the vector pointing to the nearest fish. The next movement of the $\textit{i}\text {th}$ fish is $\textbf{v}_{i}(t+1)$, which is the composite vector of $\textbf{v}_{s,i}(t)$ and $\textbf{v}_{n,i}(t)$. In addition, if the swarm tends to swim forward, then $\textbf{v}_{0}$ needs to be added to $\textbf{v}_{i}(t+1)$, and when the $\textit{i}\text {th}$ fish is near enough to the tube, vector $\textbf{v}_{w,i}(t)$ needs to be added to $\textbf{v}_{i}(t+1)$. (**C**) Field of view of a UAV. (**D**) Symbol description for (**B**).

Field of view of 180 degrees and beyond is often assumed for fish^20^. There have been several studies on features of fish eye^21^. Due to the influence of the field of view, the movement of the fish swarm could be influenced by the nearest fish and non-adjacent fish. What is more, a fish swarm does not need a leader. Similarly, in a modern swarm system, the field of view of the UAV depends on the specific situation of usage^22^. Each UAV has a fan-shaped field of view(Fig. 5C), similar to that of an individual fish.

This paper uses Tube A for explanation and applies its coordinates in Fig. 1A for analysis. In the analysis, each fish in the tube is represented by a number, and the research object is the $\textit{i}\text {th}$ fish. During the process, the position of the $\textit{i}\text {th}$ fish at this moment is $\textbf{p}_{i}(t)\in \mathbb {R}^{2}$. The field of view of the $\textit{i}\text {th}$ fish is denoted as $A_{i}$. The set of $N_{i}$ is a grouping of all marked numbers of other fish in $A_{i}$, $n_{f,i}$ is the number of fish in front of the $\textit{i}\text {th}$ fish within $N_{i}$, and $N_{i}$ is shown as

4 $$\begin{aligned} \begin{aligned} N_{i}&= \{j:\textbf{p}_{j}(t) \in A_{i}\}, \text {where } j \ne i, j = 1,2,\ldots ,m_{i}. \end{aligned} \end{aligned}$$

Movement of a single fish is described in a discrete time manner. The dynamic movement is displayed in the following

5 $$\begin{aligned} \begin{aligned}&\textbf{v}_{i}(t+1)=\textbf{v}_{0}+\textbf{v}_{s,i}(t)+\textbf{v}_{n,i}(t)+\textbf{v}_{w,i}(t)\\&\textbf{v}_{0}=\begin{bmatrix} {v}_{0} \\ 0 \end{bmatrix}, {v}_{0}>0\\&\textbf{v}_{s,i}(t) = 0.5k(\textbf{p}_{c}(t)-\textbf{p}_{i}(t))\\&\textbf{p}_{c}(t)=\frac{1}{n_{f,i}}\sum _{\textbf{p}_{j}(t)_{x} \ge \textbf{p}_{i}(t)_{x}}\textbf{p}_{j}(t),\quad j \in N_{i}\\&\textbf{v}_{n,i}(t) = \left\{ \begin{aligned} -\textit{k} (\textbf{p}_{n,i}(t)-\textbf{p}_{i}(t)), \quad&\textit{d}_{n,i}< d_{1}\\ \textit{k} (\textbf{p}_{n,i}(t)-\textbf{p}_{i}(t)), \quad&\textit{d}_{n,i}> d_{2}\\ 0, \quad&\textit{otherwise} \\ \end{aligned} \right. \\&\textit{d}_{n,i} = \min _{j \in N_{i}} \Vert \textbf{p}_{i}(t) - \textbf{p}_{j}(t) \Vert \\&\textbf{v}_{w,i}(t) = \left\{ \begin{aligned} b{d}_{w,i}\begin{bmatrix} -\sin \theta _{l} \\ -\cos \theta _{l} \\ \end{bmatrix},&\quad {d}_{w,i} \le d_{3},j=l\\ b{d}_{w,i}\begin{bmatrix} -\sin \theta _{r} \\ \quad \cos \theta _{r} \\ \end{bmatrix},&\quad {d}_{w,i} \le d_{3},j=r\\ 0,&\quad {d}_{w,i}> d_{3}, \\ \end{aligned} \right. \\&where \quad k>0,b>0,d_{1}>0,d_{2}>0,d_{3}>0,d_{1}<d_{2}.\\ \end{aligned} \end{aligned}$$

Each fish’s movement in the next unit of time is composed of four parts.

- $\textbf{v}_{0}$ is a constant velocity in the forward direction of the x-axis, representing the tendency of cooperatively swimming out of the tube, and each fish in the swarm has this velocity component.
- $\textbf{v}_{s,i}(t)$ is related to fish in front of the $\textit{i}\text {th}$ fish in the swarm. In the x-axis direction, the $\textit{i}\text {th}$ fish tends to follow the fish in front of it. This tendency can be described as a follower structure in swarm mode. The center of gravity of the fish in front of the $\textit{i}\text {th}$ fish in $A_{i}$ is $\textbf{p}_{c}(t)$, and $\textbf{v}_{s,i}(t)$ points from $\textbf{p}_{i}(t)$ to $\textbf{p}_{c}(t)$, which can be observed in Fig. 5B. Also, the descriptions of the icons on Fig. 5B can be observed in Fig. 5D.
- $\textbf{v}_{n,i}(t)$ is related to the nearest fish, and it is partly extracted from the Boid model^23^. In this paper, $\textbf{p}_{n,i}(t)$ is the nearest fish in $A_{i}$ from the $\textit{i}\text {th}$ fish. The variable $\textit{d}_{n,i}$ is the distance between $\textbf{p}_{i}(t)$ and $\textbf{p}_{n,i}(t)$. Here, three cases for $\textbf{v}_{n,i}(t)$ are defined: *repulsion, parallel and attraction*(Fig. 5A). When $\textit{d}_{n,i}$ is smaller than $\textit{d}_{1}$, the case is *repulsion*, and $\textbf{v}_{n,i}(t)$ points from fish $\textbf{p}_{n,i}(t)$ to $\textbf{p}_{i}(t)$. On the contrary, when $\textit{d}_{n,i}$ is larger than $\textit{d}_{1}$, the case is *attraction*, and $\textbf{v}_{n,i}(t)$ points from fish $\textbf{p}_{i}(t)$ to $\textbf{p}_{n,i}(t)$. Otherwise, the case is *parallel*, and $\textbf{v}_{n,i}(t)$ is null vector.
- To ensure that the swarm remains within the confines of the tube and avoids collisions, $\textbf{v}_{w,i}(t)$ realizes the function of collision avoidance when fish is about to hit a tube. In their moving direction, if the $\textit{i}\text {th}$ fish is closer to the left tube wall, then wall *j* represents the left tube wall. Otherwise, wall *j* represents the right tube wall; ${d}_{w,i}$ is the vertical distance from $\textbf{p}_{i}(t)$ to wall *j*; the velocity $\textbf{v}_{w,i}(t)$ is a vector with a length of $b{d}_{w,i}$, and its direction is the same to that of normal between $\textbf{p}_{i}(t)$ and wall *j*, away from wall *j*(Fig. 1B); angles $\theta _{l}$ and $\theta _{r}$ are formed by wall *j* and x-axis; $\textit{d}_{3}$ represents the closest distance the $\textit{i}\text {th}$ fish from wall *j* with no need of collision avoidance. Only when ${d}_{w,i}$ is smaller than $\textit{d}_{3}$, then $\textbf{v}_{w,i}(t)$ would not be null vector; $\textit{b}$ is a constant to adjust magnitude of the rebound.

Equation (5) corresponds to the algorithm in the simulation (Movie S9). Due to the small distance among the fish in this experiment, a situation like the inexistence of the nearest fish in the field of view was not considered. It considers the distribution of not only the nearest fish, but also the entire field of view, and assigns different weights to multiple influencing factors.

**Fig. 6:**
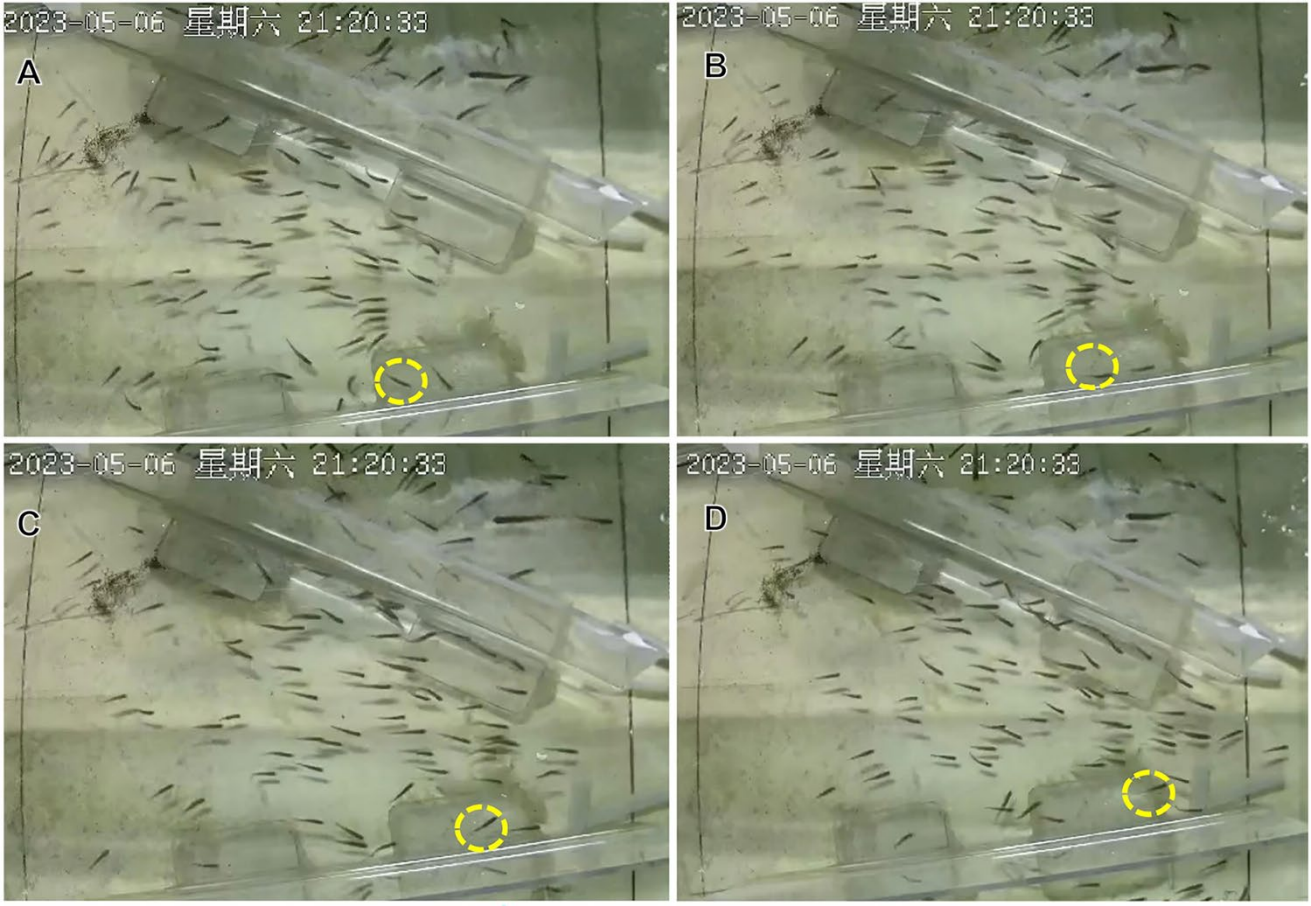
The actual motion of a specific fish when it encounters the boundary of the tube during swarm swimming, which is indicated by the circle in the picture.

From screenshots of the movie (Fig. 6), it could be found that when the $\textit{i}\text {th}$ fish was about to hit the tube, it would slow down and its motion would be added a motion vector in the opposite vertical direction to the tube. Therefore, in the simulation, when the distance between the $\textit{i}\text {th}$ fish and the tube was less than a threshold, the fish would swim for a distance along the vertical line between itself and the tube away from the tube to avoid collision. This paper applied coordinates and direction in Tube A. In their moving direction, if the $\textit{i}\text {th}$ fish is closer to the left tube wall, then the left tube wall is denoted as wall *j*. Otherwise, wall *j* represented the right tube wall. As observed in Fig. 6, when the fish drew near the tube, vector $\textbf{v}_{w,i}(t)$ pushed the fish to keep a safe distance away from wall *j*. In the simulation, $\textit{d}_{3}$ was set as 18, and $\textit{b}$ was set as 0.8.

The movement of the $\textit{i}\text {th}$ fish is affected by the nearest fish. If the nearest fish is too close, it would make adjustments to maintain proper distance. On the contrary, it would approach its neighbor if it finds itself too far away. Therefore, in *fish movement property in tubes* in this paper, three situations were considered when quantifying the behavior of the $\textit{i}\text {th}$ fish according to its distance from the nearest fish.

At the same time, since the field of view of a fish is fan-shaped, the presence of other fish within its field of view can also affect its movement. It would choose to follow the collaborative direction of the swarm, which was also taken into account in the property and represented by $\textbf{v}_{n,i}(t)$.

Because the nearest fish significantly affects the $\textit{i}\text {th}$ fish more than the rest of the swarm, the vector length corresponding to the nearest fish is more significant than that of the swarm.

**Fig. 7:**
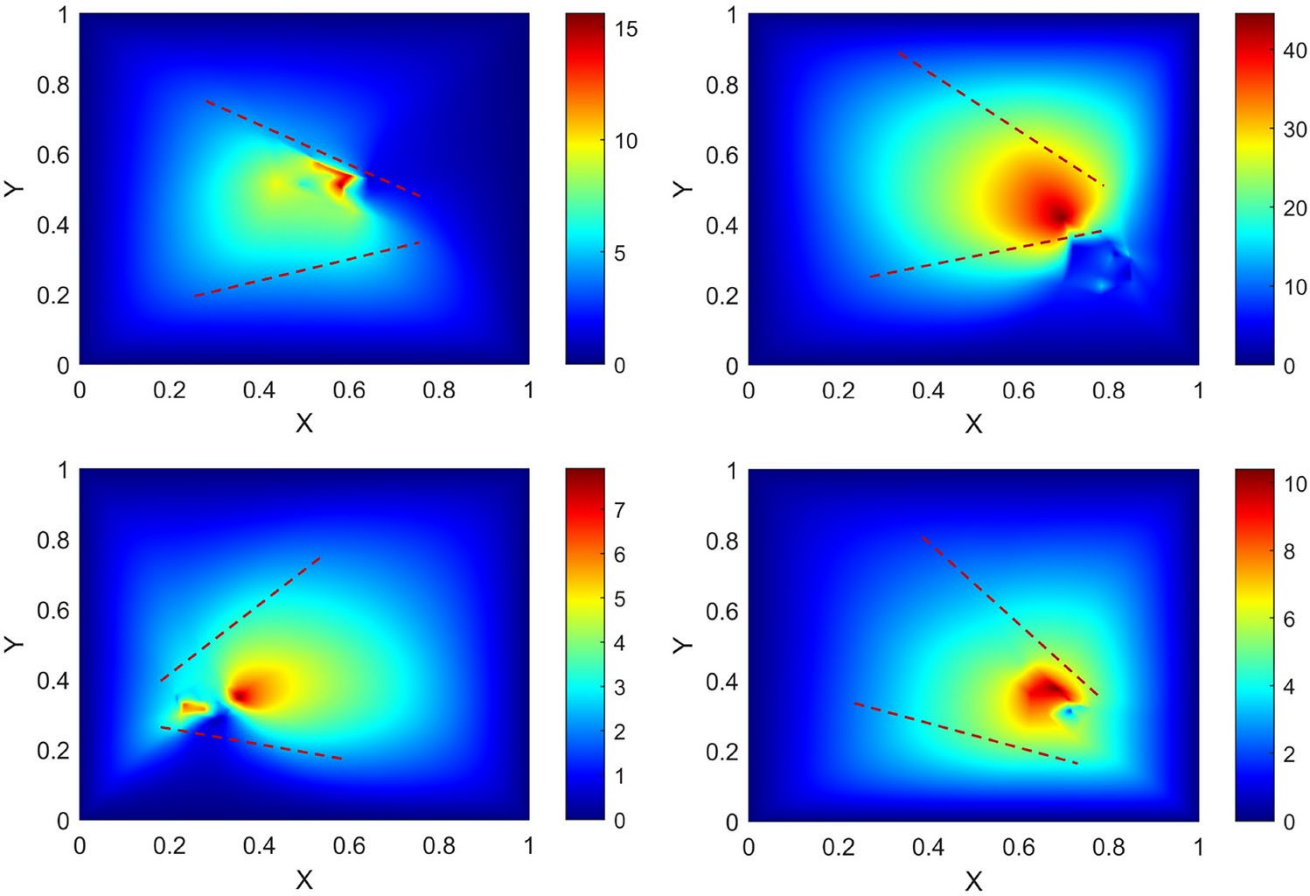
Four of the eight samples are selected here for analysis, and the locations of the tubes are drawn respectively in the four subplots. The heat map is drawn using the calculated position distribution and velocity of the fish at the moment that best corresponds to the swarming phenomenon. The color gradient, ranging from blue to red, represents the velocity value, with shades closer to red indicating higher values.

**Fig. 8:**
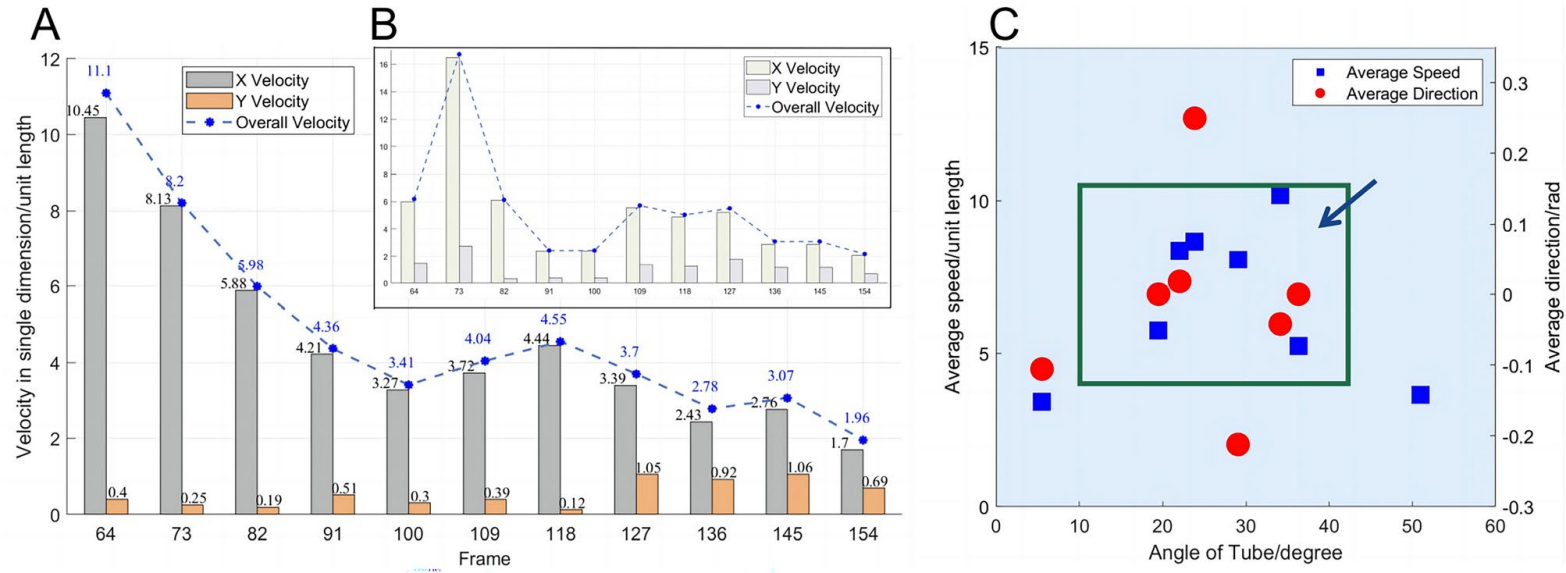
(**A**) The velocity in the x-axis and y-axis, and overall velocity of sixteen fish in Tube A over time. (**B**) The speed change of a single fish in Tube A. (**C**) Eight different tube angles and the distribution of the average velocity and direction of sixteen fish in each tube with different tube angles.

### Property 2: velocity distribution of fish swarm in tubes

There have been many studies on the distribution of the passing speed of swarm^24,25^. This paper focused on the distribution, changes in passing speed, and the influence of tube angles on speed. For tubes in different shapes, the calculation process is also different.

In Fig. 7, the fish swarm was analyzed under four different conditions using a specific frame of their respective videos (Movie S1–S8). From the image analysis, it could be concluded that at the entrance of a tube, the color representing velocity tended to be more similar to red. Most of the maximum values were in *middle region*. In contrast, in *left-edge region* and *right-edge region*, the overall speed was relatively small, so when fish swarm passed through a tube, they chose to gather in the *middle region* to achieve higher traffic efficiency.

In this paper, the average speed in *middle region* was ${V_{middle}}$, and the average speed in *edge region* was ${V_{edge}}$. Through analysis, the ratio of average speed in the two regions and the cosine of tube angle were found to form a nice linear relationship as in the following

6 $$\begin{aligned} \frac{{V_{middle}}}{{V_{edge}}}=1.5033 \cdot {\cos |\theta _{r}-\theta _{l} |} + 0.1545. \end{aligned}$$

The velocity distribution quantization took into account many factors, including the velocity ratio fitted by the software and the influence of tube angle. Meanwhile, the quantization evaded the complexity of the higher-order polynomials.

When studying how the speed of the fish change as they enter the tube, the change of the average speed over time was calculated. In Fig. 8A, Tube A was selected for analysis here. Results revealed that the expected swarm phenomenon was reached at Frame 100 of the video. It could be observed from Fig. 8B that overall velocity in the x-axis and y-axis reached a relatively small value at this moment. The movement of a single fish also corresponded to this conclusion. Concluding from the two graphs, it might be crowded when the swarm passed through a tube, and it was necessary for the swarm to reduce the speed appropriately to pass with higher efficiency.

The green box in Fig. 8C showed the position where the average speed of fish in the tube were larger on the whole. It could be observed that when the tube angle was set to approximately 22–30 degrees, the average passing speed of the swarm would increase, resulting in higher passing efficiency.

By comparing the data, this paper found that the fish swarm had a larger average speed under certain tube angles, representing higher efficiency.

### Property 3: density distribution of fish swarm in tubes

**Fig. 9:**
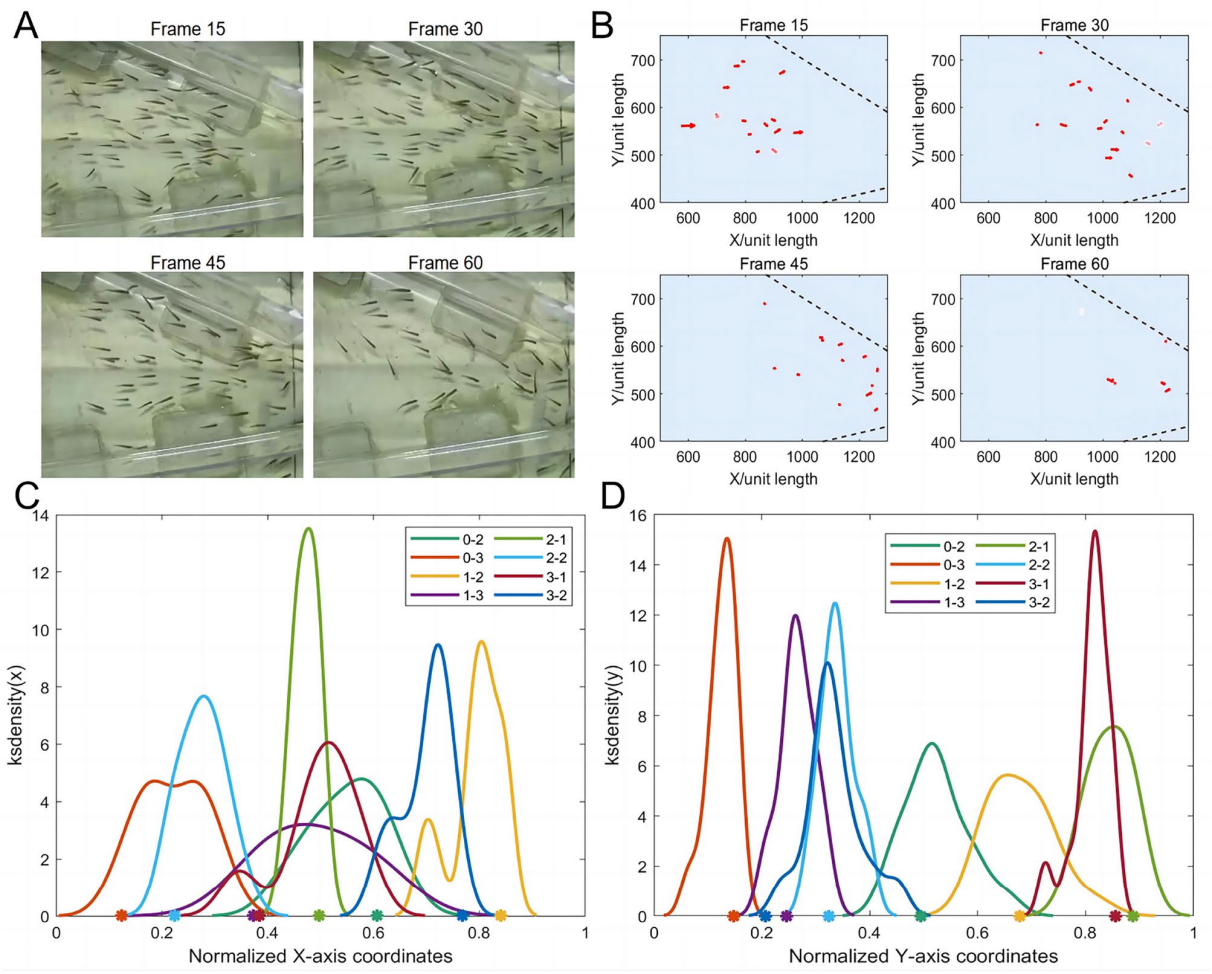
(**A**) Fish swarm distribution of Tube A at Frame 15,30,45 and 60 of its movie (Movie S1–S8) taken from an aerial perspective. (**B**) The distribution of sixteen research objects from software identification. (**C**) The probability density distribution of fish swarm numbers on the x-axis. The moment that best fits the swarm effect in each tube is selected, each color corresponds to a tube, and the symbol on the horizontal coordinate represents the position of the tube entrance. When fish swim through the tube, they prefer to gather in *middle region* instead of *edge region*. (**D**) The probability density distribution of fish swarm numbers on the x-axis, which is similar to (**C**).

**Fig. 10:**
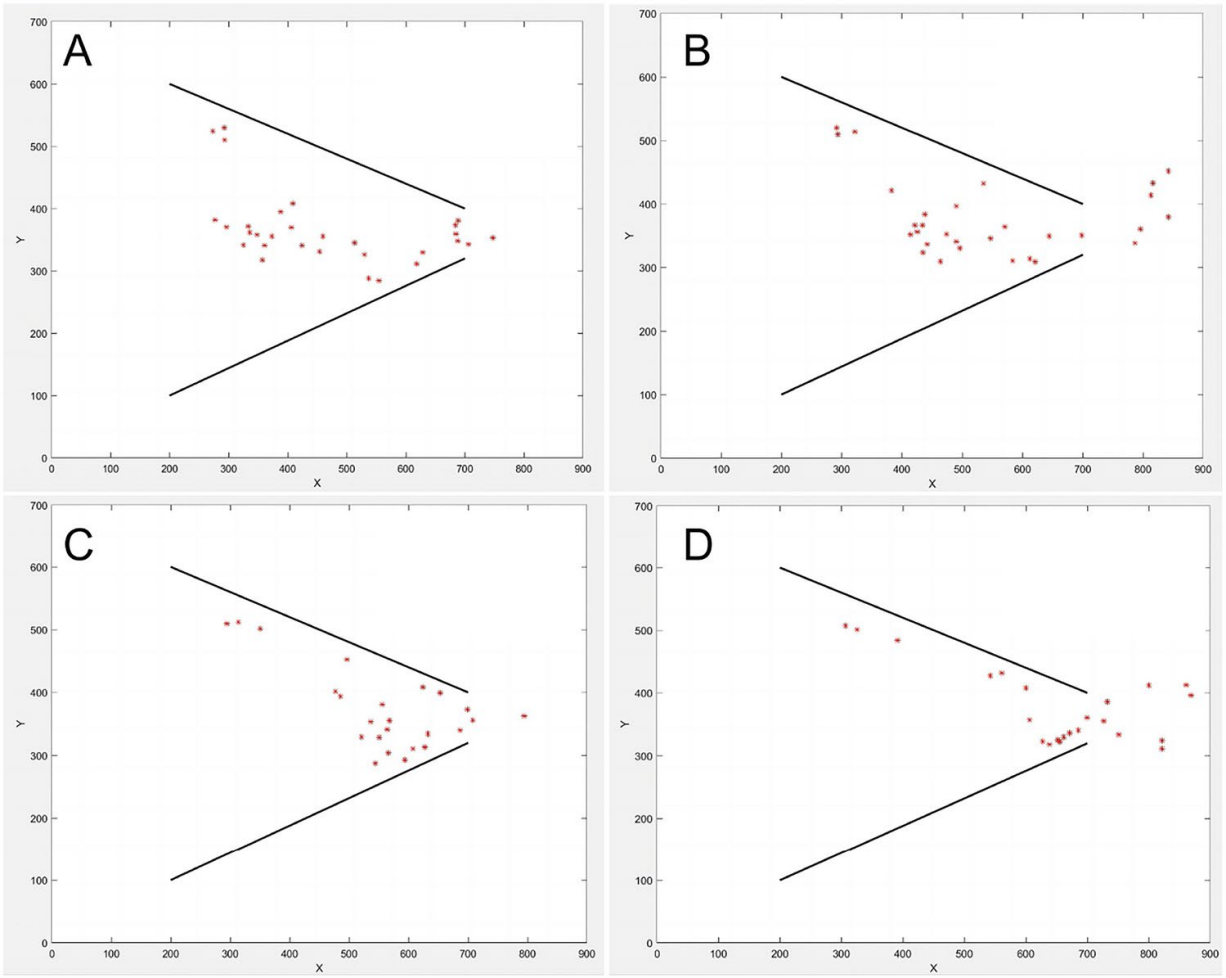
Instantaneous images extracted from the simulation of properties in this paper (Movie S9).

In order to quantify the position distribution of the swarm, this paper used the index of *ksdensity*(a function used to estimate kernel density) to describe the probability distribution of the number of fish on the x-axis and y-axis.

Fish swarm distribution of Tube A at Frame 15,30,45 and 60 of its movie (Movie S1–S8) is displayed in Fig. 9A. The distribution of sixteen research objects from software identification is displayed in Fig. 9B. Through experimental observation, it was found that for tubes in different angles, the fish swarm tended to be densely distributed in the *middle region* instead of *edge region*. From Fig. 9C,D, it could be observed that on the x-axis, peaks of the eight probability distribution curves were aligned with the points on the x-axis which represented the center of the tube entrance. The area, where fish were densely distributed, was a rectangle at the entrance of the tube. The rectangle did not spread not along the *edge region* of the tube, but the center. This conclusion verified that drawn by Ashraf et al.^17^: fish with higher speeds tended to gather in a phalanx configuration.

In this paper, the number of fish in *middle region* was denoted as ${N_{middle}}$, and the number of fish in *edge region* was denoted as ${N_{edge}}$. Through analysis, the ratio of the number of fish in these two regions and the cosine of the tube angle satisfied the exponential relationship as in the following

7 $$\begin{aligned} \frac{N_{middle}}{N_{edge}} = e^{{3.5971\cos |\theta _{r}-\theta _{l} |- 2.0173}}\quad . \end{aligned}$$

Many experiments have been conducted to study the interaction network in animal swarm^26–29^. It was found that swarm behavior of animals had an internal network for interaction and propagation, and the whole swarm tended to carry out cooperative behavior when facing sudden dangerous conditions. However, studies revealed that research objects at the edge were easy to be attacked, which would affect the safety of the swarm. This conclusion could also be verified in this paper, that fish tended to change the formation to gather in *middle region* of the tube, which could eliminate water resistance to a greater extent so that the swarm could pass with higher efficiency.

## Algorithm simulation

**Algorithm 1:**
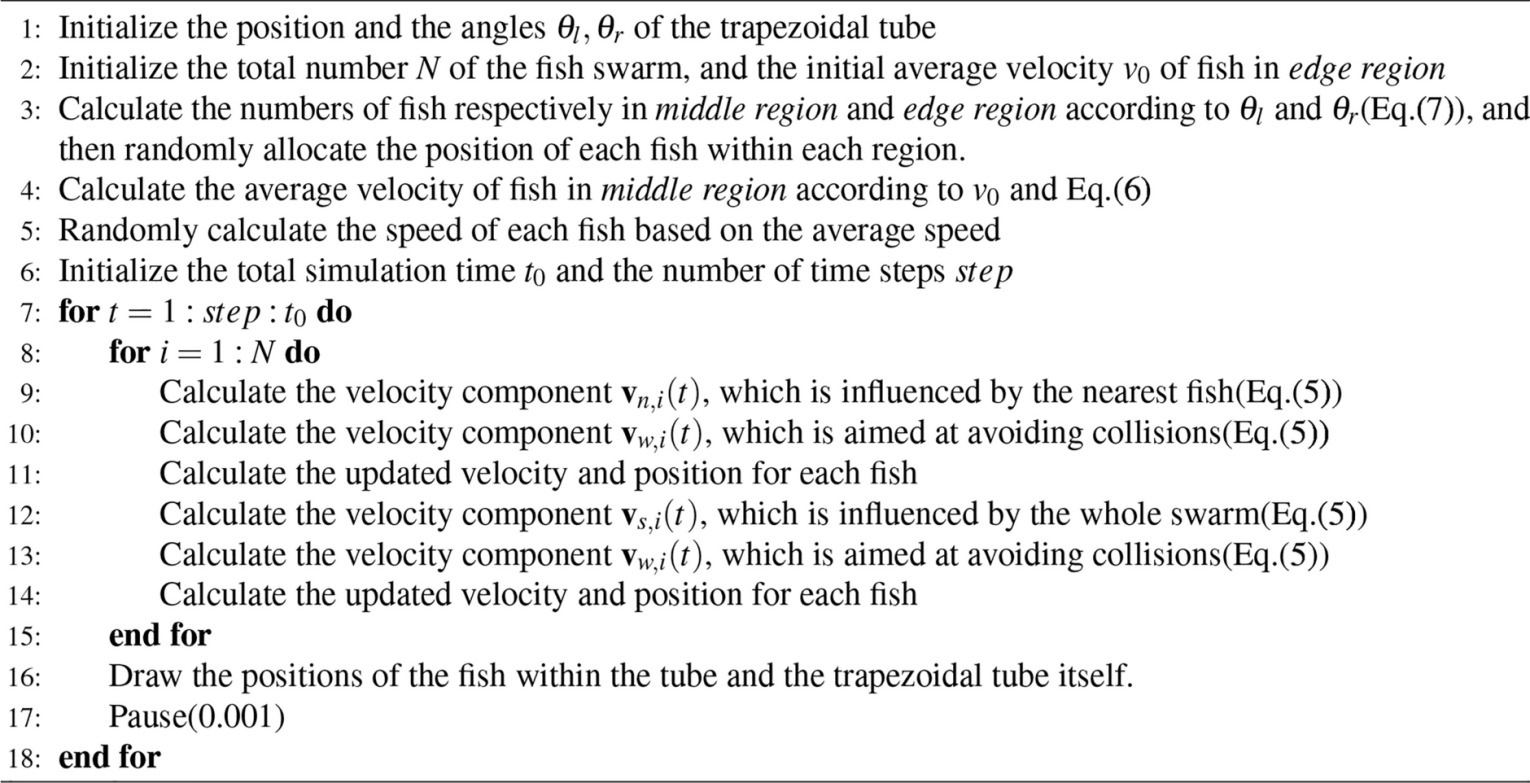
Fish movement simulation in the trapezoidal tube

### Initialization

The simulation initializes 30 fish with random positions, velocities, and directions. Fish are divided between a central zone and two side zones based on the trapezoidal geometry, with velocity and count ratios computed for each. In the simulation, this paper first set a trapezoid tube in the rectangular screen, and the inclination angle was set to correspond to the relatively high traffic efficiency achieved by the previous research. Then *middle region* and *edge region* were defined, and the number and average speed of the fish in the two regions were obtained, according to the speed ratio and density ratio in *Property 2* and *Property 3*. After that, the initial position, initial velocity, and initial azimuth of each fish were determined following the rules of random distribution. Since the premise of the simulation was that fish would exhibit the swarming phenomenon, they would move towards the outlet of the tube in the form of a swarm, so the weighting of the force driving fish away from the exit of the tube was set to be small.

### Simulation loop

At each timestep, the position of each fish is updated based on its velocity and direction. The algorithm ensures the fish maintain appropriate proximity, adjusting movement to either approach or avoid neighboring fish depending on their distance. The simulation computes the center of gravity of the fish swarm and adjusts individual fish movements based on their positions relative to the center of gravity, maintaining the overall group cohesion. Collisions with the pipe walls are detected, triggering an avoidance response to keep the fish inside the boundaries.

### Visualization

The positions of all fish are visualized in real-time as the simulation progresses, allowing for dynamic observation of the fish behavior within the constrained environment. This process balances local fish interactions with global group dynamics to simulate realistic swarm behavior.

Screen shots of the simulated movie were presented in Fig. 10.

## Discussion

In our research on the collective movement behavior of fish in narrow channels, we initially selected various fish species for observation and attempted to guide different groups using food and light stimuli. Our experiments revealed that fish capable of exhibiting pronounced and sustained collective movement under natural light conditions demonstrated stronger collective behavior. However, due to inherent differences in individual swimming characteristics, not all fish possessed the traits necessary for participating in collective swimming. As a result, we had to select individuals with distinct swimming behaviors for data analysis.

We dedicated considerable time to observing collective behavior under controlled conditions. For consistency in data analysis, we used the same group of fish during specific time periods, ensuring that variables such as light intensity and water temperature remained constant. Interestingly, we found that the collective swimming behavior exhibited a step-like pattern. The fish would suddenly begin to swim collectively at a particular moment, only to revert to a more random swimming pattern after a period of time. The chosen time window for data analysis could significantly impact our results.

We constructed eight different channel shapes for further analysis. Our findings indicated that factors such as tube angles, overall movement direction, and inter-fish distances significantly affected the speed and spatial organization of collective swimming. These results provide a crucial theoretical foundation for understanding fish behavior in tube environments.

In our study, there also existed several limitations that might influence the results. Firstly, the selected fish species have a certain body width, which could introduce bias in our findings. Secondly, while the fish were sourced from the same batch, we cannot guarantee that their response characteristics remain consistent at different moments that are not too far apart. This variability could potentially affect the overall results. Thirdly, we did not account for the impact of fin movements on water fluctuations, which might influence the final visualization outcomes. These factors could be considered in future studies to enhance the accuracy and reliability of the results.

Previous models, such as the Boid model^23^, maintained organized spatial arrangements among neighboring fish, while other models, like those proposed by Romanczuk and Lucas^30,31^, focused only on individual fish following their nearest neighbors. Our study takes a more comprehensive approach by considering both the influence of nearest neighbors and the overall distribution of the fish swarm.

Recent studies^32^ have optimized fish swarm algorithms by incorporating machine learning and deep learning techniques, including K-means, Random Forest, and LSTM. These optimized algorithms have been applied to practical problems like disease detection, contact force prediction, and urban transit network maintenance. The results of our study, combined with the latest algorithmic advancements, correspond to contemporary societal needs, particularly in areas such as robot control and road network optimization.

The properties derived from this study may inspire future algorithm development and improvements in tube design for crowded locations, potentially reducing the risk of casualties during stampedes, such as the tragic Itaewon incident in South Korea in 2022^33^. These insights are also applicable to traffic management, stampede prevention, field detection, and rescue operations.

The swarm behavior properties derived from this study have broader applications in fields such as social security, road network design, high-rise rescue operations, and pedestrian evacuation planning. Moreover, the findings can inform the development of control algorithms for robots and unmanned aerial vehicle (UAV) swarms, contributing to advances in cooperative movement theory. Numerical simulations based on these properties can improve the efficiency of fish swarms moving through narrow tubes and enhance existing models.

A particularly promising application of our findings lies in UAV swarm control^34,35^. Efficient control strategies are essential for enabling UAV swarms to perform complex tasks. By studying fish swarm behavior, researchers can develop strategies that allow UAVs to collaborate effectively in tasks such as surveillance, search and rescue, or coordinated delivery operations.

## Supplementary Information

Supplementary Information 1.

Supplementary Information 2.

## Data Availability

The datasets used and/or analyzed during the current study are available from the corresponding author on reasonable request.
