# Supplementary material for: Collective properties of Petitella georgiae in tube environments
Source: Sci Rep. 2024 Dec 2;14:29924. doi: 10.1038/s41598-024-78614-w (PMC11612465; doi:10.1038/s41598-024-78614-w)
Supplement: Supplementary file 1 — Supplementary Information 1. [file 41598_2024_78614_MOESM1_ESM.docx]

Additional reference material and data files are available at the following link: <https://rfly.buaa.edu.cn/data/statistics.rar>.These materials include 9 movies, 128 CSV files of representative swimming behavior, 79 tables of parameters in specific frames, 128 tables of total speed and direction, 8 tables of average speed, 24 tables describing location distribution and key speed in different regions, as well as Matlab codes for data processing.
