# Supplementary material for: Collective properties of Petitella georgiae in tube environments
Source: Sci Rep. 2024 Dec 2;14:29924. doi: 10.1038/s41598-024-78614-w (PMC11612465; doi:10.1038/s41598-024-78614-w)
Supplement: Supplementary file 2 — Supplementary Information 2. [file 41598_2024_78614_MOESM2_ESM.pdf]

Supplementary Material

1. Movies

1.1. Movie S1

The movies S1-S8 were cut from the original persistent movies, and this movie represented the segment during which the fish swam collectively in Tube A as a swarm.

1.2. Movie S2

This movie represented the segment during which the fish swam collectively in Tube B as a swarm.

1.3. Movie S3

This movie represented the segment during which the fish swam collectively in Tube C as a swarm.

1.4. Movie S4

This movie represented the segment during which the fish swam collectively in Tube D as a swarm.

1.5. Movie S5

This movie represented the segment during which the fish swam collectively in Tube E as a swarm.

1.6. Movie S6

This movie represented the segment during which the fish swam collectively in Tube F as a swarm.

1.7. Movie S7

This movie represented the segment during which the fish swam collectively in Tube G as a swarm.

1.8. Movie S8

This movie represented the segment during which the fish swam collectively in Tube H as a swarm.

1.9. Movie S9

This was an algorithm simulation of the three swarm models put forward in this paper.

2. Datasets

2.1. .csv files

The eight tubes in this paper were named as Tube A, B, C, D, E, F, G and H. In the following files for data processing, they corresponded to 0-2, 0-3, 1-2, 1-3, 2-1, 2-2, 3-1 and 3-2 in turn. Original statistics of trajectory recognized by the software of Zootracer were saved here.

2.2. keyframe

Here were statistics of keyframes withdrawn from the .csv files.

2.3. coordinates frame by frame

Key frames were imported into Matlab and were filed through the fitting, so positions frame by frame were gained.

2.4. parameters in specific frames

Each tube corresponded to a file, including several tables that saved the speed, position, and direction of 16 fish at this moment.

2.5. speed and direction of 16 fish in keyframes in eight tubes

In each tube, 16 fish were chosen as research objects. For instance, the table named “0208” represented the fish numbered “08” in Tube A. Speed in two dimensions and direction in keyframes of this fish were saved in this table.

2.6. average speed in each tube

Here were the average speeds of 16 fish in each tube in keyframes, and there was an additional file, which saved speeds and directions of fish in eight tubes in their own frame which fitted requirement of the swarm the best.

2.7. normalized two-dimensional position in eight tubes

Each file corresponded to a tube, saving original coordinates and normalized coordinates in the frame of the swarm of 16 fish in this tube.

2.8. speed of 16 fish in the most useful frame in eight tubes

Each file corresponded to a tube, saving speeds, direction, and normalized coordinates in the frame of the swarm of 16 fish in this tube.

2.9. speed and location in the middle region and edge region

There were eight files, respectively belonging to the eight tubes. Each file saved some tables, recording speeds, positions and directions of 16 fish at this moment. The names of the tables represented the number of frames corresponding to these statistics.
